# Association Between Trauma Mechanism and Mandibular Fracture Pattern: A 13-Year Retrospective Analysis at a Regional Trauma Center

**DOI:** 10.3390/cmtr19020022

**Published:** 2026-04-09

**Authors:** Graciela Ana Giannunzio, Jose Mariano Astigueta, Sthefania Johana Lucero, Ariana Gimena Labachuk, Carlos Alberto Isnado Bolivar

**Affiliations:** Maxilofacial Surgery, General Zonal Hospital Manuel Belgrano, Av. Constituyentes 3120, Buenos Aires 1651, Buenos Aires Province, Argentina; gra_gian@hotmail.com (G.A.G.); marianoastigueta@hotmail.com (J.M.A.); arianalabachuk6@gmail.com (A.G.L.); cbisnado93@gmail.com (C.A.I.B.)

**Keywords:** mandibular fracture, facial trauma, mechanism of injury, maxillofacial epidemiology

## Abstract

The mandible, due to its anatomical position, mobility, and functional role, is one of the bones most frequently involved in maxillofacial trauma, with fracture patterns influenced by impact mechanisms and anatomical characteristics. This study aimed to analyse the relationship between trauma mechanisms and affected anatomical subsites in patients with isolated mandibular fractures treated at a regional public hospital in Buenos Aires Province. A retrospective cross-sectional observational study was conducted using medical records, surgical reports, and diagnostic imaging of patients treated between 2011 and 2024. Isolated mandibular fractures were included, while pathological fractures, dentoalveolar injuries, and cases with incomplete data were excluded. Trauma mechanisms were classified as interpersonal aggression, vehicular accidents, falls from height, contact sports, and blows with blunt objects. Interpersonal aggression was the most frequent trauma mechanism, followed by falls from height and vehicular accidents. The mandibular angle, parasymphysis, and condyle were the most commonly affected anatomical sites. Multivariable analysis showed a higher probability of condylar fractures in falls from height (OR = 4.75; 95% CI: 2.24–10.3; *p* < 0.001) and vehicular accidents (OR = 3.02; 95% CI: 1.28–7.13; *p* = 0.01). Falls were also associated with a lower probability of mandibular angle fractures (OR = 0.16; 95% CI: 0.06–0.36; *p* < 0.001), while blunt object trauma showed a positive association with mandibular body fractures (OR = 3.12; 95% CI: 1.04–8.95; *p* = 0.04). These findings indicate that trauma mechanisms influence the anatomical distribution of mandibular fractures, providing relevant information for diagnostic assessment and surgical planning.

## 1. Introduction

The mandible is a key bone of the maxillofacial region, playing a fundamental role in chewing, swallowing, and speech, as well as being important for facial aesthetics. Its location, anatomy, and mobility make it particularly susceptible to fractures from various traumatic mechanisms. It is composed of different anatomical zones described in the AO classification [1], which categorizes them according to the affected anatomical area.

The muscles of mastication, which determine mandibular movements in the three spatial axes, attach to this bone. The elevator muscles include the medial pterygoid, masseter and temporalis; the depressor muscles are represented by the suprahyoid muscles; and the stabiliser muscles that work in the horizontal plane are the lateral pterygoids and the horizontal fibres of the temporalis. The combined action of these muscles, together with the direction and intensity of the traumatic mechanism, conditions the patterns of displacement seen in mandibular fractures [2].

Within facial trauma, the mandible and nasal bones are identified as the structures with the highest prevalence of fractures [3]. This is related to their prominent position and direct exposure to external forces, especially in contexts of assault, vehicle collisions and falls. Variable bone resistance among different craniofacial regions also influences fracture location and type. In particular, the mandible, unlike other bones of the craniofacial skeleton (CFS), is a mobile bone with active muscular insertions and exhibits complex fracture patterns that depend both on the injury mechanism and on reflex muscular tensions generated at the moment of impact.

It is known that the mechanism of trauma plays a significant role in the anatomical location of mandibular fractures [4]. For example, multiple studies indicate that total body injuries, especially those related to motorcycles, are more frequently associated with fractures in the parasymphysis or condyle region of the mandible, while interpersonal assault (IA) tends to produce fractures in the mandibular angle due to the direction and location of the impact. That is why the present article seeks to evaluate whether these associations are also evident in the population served at the Hospital Zonal General de Agudos General Manuel Belgrano (HZMB), in the district of San Martín, state of Buenos Aires, Argentina.

Unlike previous regional studies, which have primarily reported descriptive epidemiological data, the present study incorporates inferential statistical modelling to evaluate associations between trauma mechanisms and specific anatomical fracture subsites. In addition, the present cohort spans a 13-year period and focuses exclusively on isolated mandibular fractures, allowing a more precise assessment of mechanism–site relationships without confounding effects from concurrent facial fractures.

From this approach arises the following research question: Is there an association between the mechanism of trauma and the anatomical subsites of mandibular fracture among patients treated at the HZMB?

According to Ellis [5], mandibular angle fractures are among the most frequent in maxillofacial trauma. These may originate from both direct and indirect impacts, and their displacement depends on the direction, intensity and magnitude of the impact received. The author also notes that one of the most commonly observed fracture patterns in medical centres of developed countries is angle fracture combined with a contralateral body or symphyseal fracture. This combination is often attributed to IA, especially direct blows to one side of the face, which aligns with some of the patterns this research seeks to verify in this research.

In this context, the objective of the present study is to analyse the relationship between the mechanism of trauma and the anatomical subsite affected in mandibular fractures of patients treated at the HZMB, using statistical analysis to provide local evidence of a clinically and epidemiologically relevant problem, with the aim of translating this information to the general population.

## 2. Materials and Methods

A retrospective observational cross-sectional study was conducted; the information was obtained through a protocolized review of documentation from the maxillofacial surgery and traumatology department of the HZMB. Clinical records, surgical protocols of performed surgeries and radiographic, tomographic and clinical images of all facial fractures were reviewed; all images were used respecting principles of confidentiality and data anonymisation.

The research protocol was designed and conducted according to the ethical principles established in the Declaration of Helsinki and the Good Clinical Practice guidelines. Participant confidentiality and anonymity were strictly maintained throughout the study. Written informed consent was obtained from all participants prior to inclusion in the study.

From these, only cases that included the mandible were selected, and all cases with fractures in facial thirds other than the lower third, sequelae of past trauma, dentoalveolar fractures, pathological fractures, and clinical records with incomplete variables were excluded. Patients with concomitant fractures in other facial bones were also excluded to avoid confounding biomechanical interactions between multiple fracture sites, which could obscure the specific relationship between trauma mechanism and mandibular fracture location.

Data related to the number of fracture lines, subsite of fracture and mechanism of trauma attended at this institution were collected over a thirteen-year period from 2011 to 2024; data were systematised in Microsoft Excel 2021 (Microsoft Corporation, Redmond, WA, USA) for subsequent statistical analysis. This provided an integrated view of the incidence, prevalence and factors associated with facial trauma, with the purpose of relating the mechanism of trauma to the fracture site.

The cause of trauma was divided into five categories: IA, vehicle accidents (VA), contact sports (CS), falls from height (FH), and others (BBO).

IA was defined as any episode of physical violence exerted directly by one individual on another, regardless of the means used, in which the mandibular fracture was a consequence of the confrontation. In contrast, BBO was defined as mandibular fractures caused by impact with a solid object, without direct action by another person at the time of trauma.

FH were defined as vertical falls from any level, including ground-level falls and elevated falls. Vehicular accidents included both motorised and non-motorised vehicles (e.g., bicycles, motorcycles, scooters, and cars). This categorisation was chosen to preserve statistical power and avoid excessive fragmentation of subgroups with small sample sizes.

Using the data collected in Excel, the analysis was performed in R version 4.5.1 (R Foundation for Statistical Computing, Vienna, Austria). To evaluate differences in the distribution of injury mechanisms according to the anatomical site of mandibular fracture, Fisher’s exact tests were applied independently by fracture site. Each test was based on 100,000 simulations to obtain robust estimates. In addition, multivariable logistic regression models were constructed independently for each fracture site, considering mechanism of injury as the predictor variable and taking IA as the reference category, given its predominant role in the mandibular fracture caseload. Statistical significance was set at *p* < 0.05.

## 3. Results

Age ranged from 3 to 83 years, with a modal age group of 16.6–24.9 years (*n* = 222). A total of 222 patients with 356 mandibular fractures met the inclusion criteria. The cohort consisted of 193 males (86.9%), 28 females (12.6%) and 1 non-binary patient (0.5%). Demographic characteristics were relatively homogeneous across trauma mechanisms, with no clinically relevant imbalances observed. Regarding the overall distribution of fractures (Table 1), the results showed that IA was the main cause, accounting for *n* = 181 (51.1%), which constitutes not only an injury mechanism but also a manifestation of the social aetiology of facial trauma, with a particular impact on the epidemiology of mandibular fractures. IA had a significant association with fractures of the mandibular angle (*n* = 68; 37.6%; *p* < 0.001), reflecting the typical pattern of lateral impact in this type of violence. FH represented *n* = 75 (21.2%) and were predominantly associated with condylar involvement (*n* = 34; 45.3%; *p* < 0.001), an expected finding due to axial transmission of forces toward the temporomandibular joint. Blunt object (BBO) accounted for *n* = 26 (7.3%), being significantly related to fractures of the mandibular body (*n* = 7; 26.9%; *p* = 0.04), which could be attributed to the more focal nature of this mechanism of impact.

CS, although less frequent in the sample (*n* = 21; 5.9%), presented a considerable proportion of angle fractures (*n* = 6; 28.6%) and symphyseal fractures (*n* = 3; 14.3%), suggesting impact patterns both lateral and frontal. In contrast, parasymphyseal fractures were distributed more homogeneously among mechanisms (*p* = 0.18), without a statistically significant association, whereas symphyseal fractures showed a tendency towards greater involvement in sports related trauma (*n* = 3; 14.3%) and blunt object impacts (*n* = 2; 7.7%), with a *p* value close to the significance threshold (*p* = 0.05).

**Figure 1:**
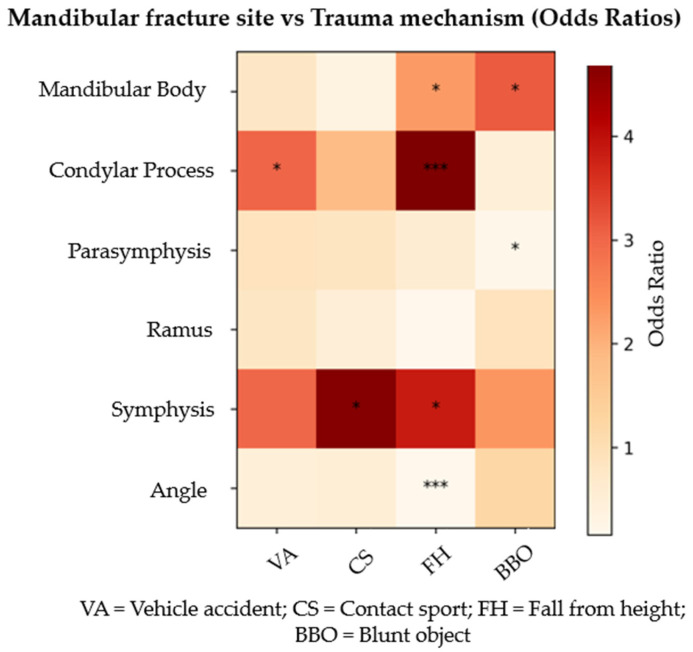
Heatmap of odds ratios (OR) for the probability of mandibular fracture at each anatomical site (vertical axis) according to mechanism of injury (horizontal axis). The analysis is based on multivariable logistic regression models by fracture site, adjusting for the different mechanisms. More intense colours indicate a stronger association between the trauma mechanism and the fracture site. Asterisks next to the heatmap cells indicate the level of statistical significance: *p* < 0.05 = *, *p* < 0.001 = ***.

The multivariable logistic regression analysis [Table 2 and Table 3, Figure 1] highlighted specific associations between mechanisms of injury and affected anatomical sites. FH were significantly associated with an increased probability of condylar fractures (OR = 4.75; 95% CI: 2.24–10.3; *p* < 0.0001) and symphyseal fractures (OR = 3.84; 95% CI: 1.2–12.69; *p* = 0.02). Conversely, this mechanism showed a lower likelihood of angle fracture (OR = 0.16; 95% CI: 0.06–0.36; *p* < 0.0001) relative to the reference group IA. BBO demonstrated a positive association with mandibular body fractures (OR = 3.12; 95% CI: 1.04–8.95; *p* = 0.04), while negative associations were observed for parasymphyseal fractures (OR = 0.20; 95% CI: 0.03–0.76; *p* = 0.04) compared with IA. Finally, VA were significantly related to condylar fractures (OR = 3.02; 95% CI: 1.28–7.13; *p* = 0.01).

These findings suggest that the traumatic mechanism conditions the anatomical fracture pattern, likely due to the direction, intensity and point of application of the force. The higher incidence of condylar fractures in FH and VA can be attributed to indirect transmission of force toward the temporomandibular joint, while mandibular body fractures associated with BBO reflect direct impact on the region. Notably, IA was the mechanism most strongly associated with mandibular angle fractures at the HZMB, a relevant finding in the characterisation of this type of trauma.

## 4. Discussion

Several studies conducted in Latin America have addressed the epidemiology of mandibular fractures, focusing both on causes of trauma and the most frequently affected anatomical locations. Below, the findings of three relevant studies carried out in Venezuela, Brazil and Chile are described, which allow comparisons with the population treated at the HZMB in Argentina.

The patients’ ages ranged from 3 to 83 years, with the highest frequency observed in the 16.6–24.9-year age group (*n* = 222). This distribution is clinically relevant because skeletal maturity, mandibular morphology, and dentition status vary across age groups and may influence trauma biomechanics and fracture patterns. The predominance of fractures in young adults is consistent with epidemiological studies reporting peak incidence in the second and third decades of life, a period associated with greater exposure to interpersonal violence, traffic accidents and high-energy activities. Moreover, mandibular fractures are generally more common in younger populations compared with older adults, likely reflecting behavioural and social risk factors as well as differences in bone resilience and trauma mechanisms. These aspects should be considered when interpreting fracture patterns and planning preventive strategies.

In 2017, Amarista-Rojas et al. [6] analysed 334 patients with a total of 522 mandibular fractures. Regarding aetiology, motorcycle vehicle accidents were the most common cause (28.1%). The most affected anatomical site was the parasymphyseal region, representing 27.6% of the fractures. In addition, combined fractures were reported, representing 42.32% of patients with two fracture lines, 7.4% with three fracture lines and finally 0.52% with four fracture lines. These results suggest a strong relationship between high-energy mechanisms, such as motorcycle accidents, and fractures in the anterior region of the mandible, as well as a tendency to affect multiple anatomical regions simultaneously [6].

In 2015, Marinho et al. [7] conducted a study that included 171 patients and a total of 269 mandibular fractures. The most frequent causes were motorcycle accidents and accidental falls. Regarding anatomical distribution, the mandibular condyle was the most affected site (32.04%), followed by the mandibular angle (23.38%). This study suggests that different trauma mechanisms can condition fracture patterns, with an association observed between VA and condylar fractures, while other mechanisms, such as interpersonal assault (IA), could be more linked to the mandibular angle region [7].

In 2024, Céspedes Arroyo et al. [8] analysed 42 surgical cases with a total of 62 mandibular fractures between 2020 and 2022. In this study, IA was identified as the most common cause of mandibular fracture, representing 73.8% of cases. The mandibular angle was the most frequently affected anatomical region, with a frequency of 40.35%. These data support the hypothesis that direct interpersonal assaults tend to produce fractures at the mandibular angle, a region vulnerable to lateral impacts, especially when there is no defensive or protective action [8].

The strong association between interpersonal assault and angle fractures may reflect typical lateral impact vectors, frequently delivered by a dominant hand, which concentrate force on the mandibular angle. Conversely, mechanisms involving axial loading, such as falls and vehicular accidents, tend to transmit forces posteriorly toward the condyle. These biomechanical considerations support the statistical associations observed.

Additionally, studies focusing on triple mandibular fractures have highlighted the role of high-energy trauma, such as motor vehicle accidents, in generating multifocal fracture patterns. These injuries commonly combine anterior mandibular fractures with condylar or ramus involvement, which may be explained by biomechanical force transmission across the mandible. Such findings reinforce the hypothesis that trauma mechanism significantly influences both the anatomical site and the complexity of mandibular fractures [9].

In contrast to some Latin American series in which vehicular accidents were the predominant mechanism, interpersonal assault was the leading cause in our cohort. This discrepancy may reflect regional sociocultural differences, urban violence patterns, and variations in inclusion criteria, particularly our restriction to isolated mandibular fractures.

Although several epidemiological studies have described the distribution of mandibular fractures and their causes, many have focused primarily on descriptive analyses without exploring the statistical association between trauma mechanisms and specific fracture subsites. In this context, the present study contributes additional evidence by applying inferential statistical analysis to evaluate these relationships. Our findings therefore complement previous regional studies by providing statistical support to clinical observations that have traditionally been described in descriptive terms.

This study presents certain limitations that should be considered when interpreting the results. First, the analysis was restricted to isolated mandibular fractures, excluding patients with associated facial fractures. While this approach allowed a more specific evaluation of the relationship between trauma mechanisms and mandibular fracture sites, it may limit the generalisability of the findings to patients with complex maxillofacial trauma. Second, some trauma mechanisms of different energy levels were grouped into broader categories during data analysis. For example, bicycle accidents were analysed together with other vehicular accidents such as car or motorcycle collisions. Although this grouping facilitated statistical comparison, it may introduce a degree of heterogeneity in the trauma energy involved and could potentially influence the observed associations.

Overall, the reviewed literature indicates that the traumatic mechanism plays a significant role in the anatomical location of mandibular fractures. However, these studies lack statistical analysis to support these findings. Clinically, understanding mechanism-specific fracture patterns may assist surgeons in directing imaging evaluation, anticipating associated injuries, and prioritising surgical approaches, particularly in emergency settings where full radiological assessment may not yet be available.

## 5. Conclusions

This study indicates that the mechanism of injury can be a determinant factor in the localization of mandibular fractures. This information may be useful for clinical diagnosis and therapeutic planning in patients with maxillofacial trauma. It is recommended to consider these patterns during the initial assessment, as they may also support clinical suspicion and targeted surveillance for occult mandibular fractures according to trauma etiology. Further studies integrating sociodemographic variables and trauma characteristics are encouraged to deepen understanding of these associations.

Finally, this study provides an important basis for future research aimed at expanding and consolidating knowledge about the factors that determine the distribution of mandibular fractures.

## Figures and Tables

**Table 1:** Distribution of mandibular fractures by anatomical site according to different mechanisms of injury, with Fisher’s exact test *p*-values for each fracture site.

Fracture Site	VA	CS	FH	IA	BBO	*p* Value
Angle	13 (25.5%)	6 (28.6%)	7 (9.3%)	68 (37.6%)	11 (42.3%)	<0.001
Mandibular Body	5 (9.8%)	1 (4.8%)	13 (17.3%)	21 (11.6%)	7 (26.9%)	0.04
Condylar Process	13 (25.5%)	5 (23.8%)	34 (45.3%)	25 (13.8%)	2 (7.7%)	<0.001
Parasymphysis	13 (25.5%)	5 (23.8%)	12 (16%)	46 (25.4%)	2 (7.7%)	0.18
Ramus	3 (5.9%)	1 (4.8%)	2 (2.7%)	15 (8.3%)	2 (7.7%)	0.40
Symphysis	4 (7.8%)	3 (14.3%)	7 (9.3%)	6 (3.3%)	2 (7.7%)	0.05
Total	51 (14.4%)	21 (5.9%)	75 (21.2%)	181 (51.1%)	26 (7.3%)	

VA = Vehicle accident; CS = Contact sport; FH = Fall from height; IA = Interpersonal assault; BBO = Blunt object.

**Table 2:** Results of multivariable logistic regression models, where the dependent variable is the occurrence of fracture at a specific mandibular site, and the independent variables are mechanisms of injury (compared with the reference category “IA”).

Fracture Site	Mechanism of Injury	Coefficient (β)	Standard Error (SE)	Z	*p* Value	OR (95% CI Lower–Upper)
Mandibular Body						
	VA	0.78	0.59	−0.41	0.68	0.78 (0.21–2.3)
	CS	0.35	1.06	−0.99	0.32	0.35 (0.02–1.9)
	FH	2.28	0.42	1.98	0.05	2.28 (0.99–5.12)
	BBO	3.12	0.54	2.1	0.04	3.12 (1.04–8.95)
Condylar Process						
	VA	3.02	0.44	2.54	0.01	3.02 (1.28–7.13)
	CS	1.86	0.59	1.05	0.30	1.86 (0.54–5.75)
	FH	4.75	0.39	4.03	<0.0001	4.75 (2.24–10.3)
	BBO	0.47	0.78	−0.98	0.33	0.47 (0.07–1.78)
Parasymphysis						
	VA	0.85	0.43	−0.36	0.72	0.85 (0.35–1.97)
	CS	0.81	0.58	−0.36	0.72	0.81 (0.24–2.44)
	FH	0.59	0.4	−1.3	0.19	0.59 (0.26–1.27)
	BBO	0.2	0.77	−2.06	0.04	0.2 (0.03–0.76)
Ramus						
	VA	0.79	0.67	−0.35	0.73	0.79 (0.17–2.63)
	CS	0.49	1.07	−0.66	0.51	0.49 (0.03–2.73)
	FH	0.17	1.05	−1.68	0.09	0.17 (0.01–0.89)
	BBO	0.86	0.8	−0.19	0.85	0.86 (0.13–3.44)
Symphysis						
	VA	2.99	0.68	1.6	0.11	2.99 (0.72–11.26)
	CS	4.67	0.77	2	0.05	4.67 (0.9–20.28)
	FH	3.84	0.59	2.28	0.02	3.84 (1.2–12.69)
	BBO	2.33	0.86	0.99	0.32	2.33 (0.32–11.18)
Angle						
	VA	0.47	0.43	−1.8	0.07	0.47 (0.2–1.06)
	CS	0.51	0.56	−1.21	0.23	0.51 (0.16–1.5)
	FH	0.16	0.45	−4.08	<0.0001	0.16 (0.06–0.36)
	BBO	1.2	0.52	0.35	0.73	1.2 (0.44–3.45)

VA = Vehicle accident; CS = Contact sport; FH = Fall from height; IA = Interpersonal assault; BBO = Blunt object.

**Table 3:** Summary of significant (or near-threshold) associations identified by logistic regression between different mechanisms of injury and mandibular fracture sites.

Mechanism of Injury	Most Probable Fracture Site(s)	Odds Ratio (95% CI)	*p* Value
FH	Condylar Process	4.75 (2.24–10.3)	<0.0001
	Mandibular Body	2.28 (0.99–5.12)	0.05
	Symphysis	3.84 (1.2–12.69)	0.02
	Angle	0.16 (0.06–0.36)	<0.0001
BBO	Mandibular Body	3.12 (1.04–8.95)	0.04
	Parasymphysis	0.20 (0.03–0.76)	0.04
VA	Condylar Process	3.02 (1.28–7.13)	0.01
CS	Symphysis	4.67 (0.9–20.28)	0.05

VA = Vehicle accident; CS = Contact sport; FH = Fall from height; BBO = Blunt object.

## Data Availability

The original contributions presented in this study are included in the article. Further inquiries can be directed to the corresponding author.

## Abbreviations

The following abbreviations are used in this manuscript:

IA	Interpersonal assault
VA	Vehicle accident
CS	Contact sport
FH	Fall from height
BBO	Blunt object
HZMB	Hospital Zonal General de Agudos General Manuel Belgrano
OR	Odds ratio
CI	Confidence interval
CFS	Craniofacial skeleton
